# Emotion recognition deficits in patients with skin picking disorder: the role of alexithymia while controlling depression, and anxiety levels

**DOI:** 10.3389/fpsyt.2025.1597268

**Published:** 2025-05-16

**Authors:** Mine Ergelen, Aliye Canan Taşlıoğlu Sayıner, Mustafa Çırakoğlu, Murat Yalçın

**Affiliations:** ^1^ Istanbul Erenkoy Training and Research Hospital for Psychiatric and Neurological Diseases, Istanbul, Türkiye; ^2^ Faculty of Economics, Administrative and Social Sciences, Department of Psychology, Bahçeşehir University, Istanbul, Türkiye; ^3^ School of Medicine, Marmara University, Istanbul, Türkiye

**Keywords:** skin picking disorder, alexithymia, emotion recognition, body-focused repetitive behavior disorder, depression, anxiety

## Abstract

**Objective:**

Skin Picking Disorder (SPD) has been associated with higher levels of alexithymia, a condition that predicts self-injurious behaviors, a core feature of SPD. Recent studies have expanded the understanding of alexithymia beyond deficits in emotional awareness, highlighting its role on the ability to recognize and process others’ emotions. This study aimed to explore how emotion recognition abilities differ in individuals with varying levels of alexithymia and the presence of SPD.

**Methods:**

This cross-sectional case-control study included 45 individuals diagnosed with SPD and 47 controls. Participants were assessed through the Toronto Alexithymia Scale, the Facial Emotion Recognition Test, the Beck Depression Inventory, and the Beck Anxiety Inventory. Two-way analysis of covariance tests were conducted to evaluate the effects of SPD diagnosis and alexithymia levels on facial emotion recognition tasks, while controlling for anxiety and depression.

**Results:**

Individuals with Skin Picking Disorder (SPD) showed significant impairments in recognizing fear, neutral expressions, and surprise compared to controls. SPD was linked to lower fear recognition accuracy, while both SPD and higher alexithymia were associated with poorer recognition of neutral expressions and surprise. No significant differences were found for happiness, sadness, anger, or disgust. No interaction effects were observed between SPD and alexithymia for any emotion.

**Conclusions:**

This study enhances the understanding of emotion recognition in SPD and alexithymia by highlighting their shared and unique challenges. The absence of a significant interaction effect suggests that these conditions independently contribute to emotion recognition deficits without compounding effects, underscoring the need for targeted interventions.

## 1 Introduction

Skin-picking disorder (SPD) is a body-focused repetitive behavior characterized by recurrent and compulsive picking of the skin, which results in skin lesions and can lead to significant distress or impairment in daily functioning. Originally, SPD was classified under impulse control disorders not otherwise specified in the Diagnostic and Statistical Manual of Mental Disorders- Text Revision, Fourth Edition (DSM-IV-TR) due to its association with impulsive behaviors (1). However, with the release of the Diagnostic and Statistical Manual of Mental Disorders, Fifth Edition (DSM-5), SPD was reclassified under obsessive-compulsive and related disorders (2). This shift in classification highlights the compulsive nature of SPD, aligning it more closely with disorders such as trichotillomania, hoarding disorder, body dysmorphic disorder, and obsessive-compulsive disorder. These conditions share a common theme of repetitive behaviors and mental preoccupations that are often driven by an underlying urge or compulsion, rather than an impulsive act alone. For instance, the act of skin-picking in patients with SPD is often driven by an overwhelming urge that arises from an internal sense of tension or emotional discomfort. The compulsive behavior serves as a temporary relief from these negative feelings, yet it simultaneously leads to non-suicidal self-injury, manifesting as skin lesions and scarring (3).

Research indicates that SPD is associated with negative emotions, low self-esteem, reduced subjective physical well-being, and a loss of control (4). A significant proportion of individuals with SPD have at least one lifetime comorbid psychiatric disorder, with anxiety and depression being the most prevalent ones. The prevalence of depression among individuals with SPD ranges from 12.5% to 48%, while anxiety disorders are reported in 8% to 23% of cases (5). Additionally, in a study of 92 individuals diagnosed with SPD, 85.9% reported experiencing anxiety, and 66.3% reported depression as a result of their skin picking behaviors (6). In Turkish clinical samples, the psychiatric comorbidity rate among SPD patients was found to be 78.9% (7).

Alexithymia is a psychological construct characterized by difficulty in identifying and describing emotions, distinguishing emotions from bodily sensations, and a tendency toward externally oriented thinking (8). First introduced by Sifneos (9), the concept emerged within the field of psychosomatic medicine and was initially viewed as a personality trait that increased vulnerability to psychosomatic illness (10). Beyond its established role in psychosomatic conditions (e.g., 11, 12), alexithymia has been widely studied across various psychiatric conditions. It has been linked to anxiety (13), obsessive-compulsive disorders (14, 15), eating disorders (16), personality disorders (17), depressive disorders in psychiatric patients (18).

Recent evidence has also emphasized its role as a significant vulnerability factor in the onset and progression of chronic medical conditions and increasingly been studied in relation to diseases such as asthma, inflammatory bowel disease and type 2 diabetes (19, 20) and psychodermatological disorders such as alopecia areata, psoriasis and chronic urticaria (21). Research indicates that individuals with high levels of alexithymia often exhibit impairments in emotion regulation, increased psychological distress, and reduced adherence to treatment—factors that may contribute to poorer health outcomes and increased disease severity (22, 23). It has also been associated with avoidant coping strategies and immature defense mechanisms, such as denial, particularly in response to emotional stress and trauma (24, 25). These defensive processes may offer short-term protection but can impair the integration of memory, perception, and emotion into conscious awareness (26, 27). Alexithymia is also a significant predictor of non-suicidal self-injury (28), with evidence suggesting that individuals with alexithymia may engage in self-injury to manage their emotional states (29). This connection extends to body-focused repetitive behaviors, which are considered maladaptive strategies for emotion regulation. According to the emotion regulation model, body-focused repetitive behaviors are triggered by negative emotions and provide short-term relief by alleviating unpleasant feelings, though they ultimately lead to increased emotional and physiological distress (30). Additionally, individuals with skin-picking disorder (SPD) exhibit altered neural sensitivity in touch processing, which has been linked to emotional regulation deficits. This disrupted sensory experience may reinforce maladaptive self-soothing behaviors, further contributing to SPD symptoms (31).

Recent research has expanded beyond understanding alexithymia as merely an emotion awareness deficit, showing its relationship with the ability to recognize and process others’ emotions. Clinical studies have demonstrated that individuals with alexithymia also experience deficits in recognizing facially expressed emotions of others (32). Even among non-clinical populations, those with high alexithymia show impairments in recognizing facially expressed emotions. During facial emotion recognition tests, alexithymic individuals exhibit lower activation in brain areas associated with emotional awareness (such as the anterior cingulate cortex) and regions involved in facial emotion recognition (including the amygdala, insula, striatum, inferior frontal gyrus, middle temporal gyrus, thalamus, parahippocampal gyrus, and middle frontal gyrus) compared to non-alexithymic individuals (33). A recent neuroimaging study highlighted that the cognitive dimension of alexithymia is particularly associated with lower activation in emotional attention and recognition networks, leading to deficits in emotion processing (34).

Also, SPD has been increasingly linked to deficits in facial emotion recognition (35), a fundamental aspect of social cognition essential for effective interpersonal interactions and emotional regulation. These impairments may contribute to increased emotional distress by limiting an individual’s ability to interpret and respond to social cues, reducing the effectiveness of interpersonal interactions as a means of emotional regulation. Consequently, individuals with SPD may rely more heavily on maladaptive coping mechanisms, such as compulsive skin-picking, to manage distress (36). Furthermore, the lower capacity for emotional self-awareness and insight, further impairing individuals’ ability to recognize and address triggers underlying their compulsive behaviors (34). As a result, alexithymia may play a significant role in the persistence of SPD symptoms by reinforcing the cycle of emotion dysregulation and self-injurious behavior. However, the extent to which emotion recognition impairments in SPD are linked to alexithymia, or whether they occur independently, remains an open question that has not been adequately explored in the literature.

### 1.1 Objectives of the study

Despite the high prevalence of alexithymia in many psychiatric disorders (37) there is a limited research specifically examining the interplay between alexithymia and facial emotion recognition in SPD. While previous studies have investigated emotion recognition and alexithymia separately across different psychiatric populations, few have explored their interaction in individuals with SPD. Aydin et al. (35) demonstrated that individuals with SPD and Trichotillomania exhibit increased alexithymia. In addition, Kłosowska et al. (38) found that individuals with SPD reported reduced emotional awareness and lower interoceptive accuracy—both traits associated with alexithymia—suggesting difficulties in associating internal bodily cues with emotional states. Similarly, Ciuluvica et al. (39) found that patients with chronic skin conditions exhibited higher levels of emotion suppression and alexithymic traits compared to healthy controls, with significant associations between emotion regulation deficits and poorer quality of life.

In light of these research, this study aims to fill the critical gap by comprehensively assessing facial emotion recognition abilities in individuals with SPD, comparing their performance with that of controls while accounting for varying levels of alexithymia. Importantly, by controlling for depression and anxiety—two conditions highly comorbid with SPD—we aim to disentangle the specific contribution of alexithymia to emotion recognition deficits and determine whether such impairments reflect an intrinsic feature of SPD or are secondary to broader affective symptoms

By addressing these questions, our study may provide novel insights into the underlying cognitive and emotional mechanisms contributing to SPD. A clearer understanding of these factors could have significant clinical implications, guiding the development of targeted interventions aimed at improving emotion recognition and regulation in individuals with SPD. If facial emotion recognition deficits are found to be closely tied to alexithymia, treatment approaches incorporating emotional awareness training may be particularly beneficial. Conversely, if such deficits exist independently of alexithymia, interventions may need to focus more directly on enhancing social cognition skills in SPD patients.

## 2 Method

### 2.1 Sampling

This cross-sectional case-control study, conducted between February and August 2024, included 45 patients with SPD diagnosis from dermatology outpatient units of Marmara University Medical Faculty and Erenköy Training and Research Hospital for Mental and Neurological Diseases. A dermatology specialist recorded medical histories. Eligible participants were adults with SPD, without comorbid psychotic disorders or cognitive deficits, and not receiving psychiatric treatment in the past year due to its impact on emotion recognition (40). Psychiatric examinations were conducted at Erenköy Training and Research Hospital for Mental and Neurological Diseases. The control group comprised age- and gender-matched volunteers from the hospital’s administrative staff, excluding those with skin diseases, chronic illnesses, psychosis, cognitive deficits, or recent psychiatric treatment. Ethical approval was granted by Marmara University, Faculty of Medicine (09.02.2024/219), and written informed consent was obtained from all participants.

### 2.2 Measures

All participants filled out the sociodemographic form, the Toronto Alexithymia Scale, the Ekman Emotion Recognition Scale, the Beck Anxiety Inventory, and the Beck Depression Inventory.

*Sociodemographic Form:* This form, prepared by the researchers, contained questions regarding the participants’ age, gender, education, and marital status.

*Toronto Alexithymia Scale-20 (TAS-20)*: The Toronto Alexithymia Scale (8) consists of 20 items in three subscales, namely the Difficulty Identifying Feelings (DIF), Difficulty Describing Feelings (DDF), and Externally-Oriented Thinking (EOT). Each item is rated on a five-point Likert scale (1-5), with total scores ranging from 20 to 100. The validation study of its Turkish form was conducted by Güleç et al. (41), where a score of 60 or above on the scale indicates the presence of severe alexithymia. The Cronbach’s alpha coefficient of the scale was.77 in the present study.

*Facial Emotion Recognition Test (FERT):* The photographic set utilized in this research, compiled by Ekman and Friesen (42), comprises images depicting six distinct emotional expressions—happiness, sadness, fear, anger, surprise, and disgust—alongside a neutral expression. The set includes a total of 56 photographs representing 8 models, four females and four males. Initially, patients were introduced to the first seven trial photographs; subsequently, they were requested to identify the remaining 49 images. The photographs were displayed to the patients on a computer in a slideshow format (43), and they were required to respond within a timeframe of 5 seconds.

*Beck Depression Inventory (BDI): BDI* (44, 45) is used to measure the level of depressive symptoms in adults. It consists of 21 items, each scored from 0 to 3, allowing for a total score range of 0 to 63. A cutoff score of 17 has been established for severe depression. The Turkish validity and reliability of this scale have been conducted by Hisli (46).

*Beck Anxiety Inventory (BAI):* BAI (47) consists of twenty-one items, each scored on a Likert scale from 0 to 3. This scale is specifically designed to evaluate the severity of both subjective and somatic symptoms of anxiety in individuals. The Turkish adaptation of this scale has been validated and tested by Ulusoy et al. (48), confirming its suitability and efficacy for use within the Turkish population.

### 2.3 Statistical analysis

Data analysis was conducted using SPSS for Windows version 24.0. The normality of the Difficulty Identifying Feelings (D(92) = .058, p = .900), Difficulty Describing Feelings (D(92) = .088, p = .073), and Externally-Oriented Thinking (D(92) = .083, p = .140) subscales of the TAS-20 was assessed using the Kolmogorov–Smirnov test. Additionally, the normality of the Happiness (D(92) = .117, p = .151), Sadness (D(92) = .077, p = .614), Fear (D(92) = .070, p = .724), Surprise (D(92) = .120, p = .131), Disgust (D(92) = .076, p = .629), and Neutral (D(92) = .069, p = .753) subscales of the Facial Emotion Recognition Test, as well as scores from the BDI (D(92) = .121, p = .127) and the BAI (D(92) = .069, p = .744), were also examined.

Between-group differences in continuous variables (e.g., age, TAS-20, FERT, BDI, and BAI scores) were analyzed using independent samples t-tests. Pearson correlation analyses were performed to examine relationships between alexithymia, depression, anxiety, and emotion recognition performance. A series of two-way analysis of covariance (ANCOVA) tests were performed to investigate the main and interaction effects of SPD diagnosis (SPD vs. control) and alexithymia levels (low vs. high) on emotion recognition performance as measured by the Facial Emotion Recognition Test (FERT). Given the significant differences in anxiety and depression levels between the groups, these variables were included as covariates in all ANCOVA models to control for their potential confounding effects. Separate ANCOVA tests were conducted for each emotion category (happiness, sadness, fear, anger, surprise, disgust, and neutral expressions).

*A priori* power analysis for ANCOVA was conducted using G*Power 3.1 software to determine the required sample size for detecting medium effect sizes (f = 0.25) with a power of 0.80 and an alpha level of 0.05. The results indicated that a minimum total sample size of 88 participants was necessary to achieve sufficient statistical power. The current study’s sample size (N = 92) met this requirement, ensuring adequate power to detect meaningful effects.

## 3 Results

### 3.1 Demographic variables of participants

The study included 92 participants, consisting of 45 individuals diagnosed with SPD and 47 individuals in the control group. The SPD group consisted of 29 females (64.4%) and 16 males (35.6%), while the control group included 29 females (61.7%) and 18 males (38.3%). An independent sample t-test was performed to determine whether there was a significant difference between the mean age of participants diagnosed with SPD and those in the control group. The results indicated that participants with SPD (M = 42.18, SD = 11.82) and control group participants (M= 38.49, SD = 10.85), did not significantly differ from each other, t(90) = 1.56, p = .122.

Furthermore, 15 participants (31.91%) of the SPD group had comorbid depressive disorders, 4 participants (8.51%) had anxiety disorders, and 11 participants (23.40%) had both depressive and anxiety disorders. Additionally, 1 participant (2.13%) had obsessive-compulsive disorder (OCD), and 2 participants (4.4%) had dissociative disorders. Meanwhile, 12 participants (25.53%) of the SPD group had no comorbid psychiatric diagnosis history, whereas all participants in the control group reported no history of psychiatric diagnosis within the past year. All demographic characteristics of the sample can be found in **Table 1**.

**Table 1:** Characteristics of the sample.

Variable	SPD		Control	
	n	%	n	%
Gender				
Female	29	64.4	29	61.7
Male	16	35.6	18	38.3
Education				
Primary/secondary school	23	51.1	20	42.5
High school	14	31.1	17	36.2
Vocational school/undergraduate degree	7	15.6	10	21.3
Graduate degree	1	2.2	–	–
Relationship Status				
Single	7	15.6	8	17.1
Married	34	75.6	34	72.3
Widowed/Separated	4	8.8	5	10.6
Comorbid Psychiatric Diagnosis				
No diagnosis	12	25.53	47	100
Depressive Disorders (DD)	15	31.91	–	–
Anxiety Disorders (AD)	4	8.51	–	–
DD + AD	11	23.40	–	–
OCD	1	2.13	–	–
Dissociative Disorders	2	4.26	–	–

*N*= 92., SPD: Skin Picking Disorder Group.

When the groups were compared regarding their scale scores (**Table 2**), the SPD group displayed a significantly higher level of BAI (*M_diff_* = 10.561, *t*(50.91) = 6.072, *p* <.001), BDI (*M_diff_* = 13.696, *t*(55.58) = 5.374, *p* <.001) and TAS -20 (*M_diff_* = 5.192, *t*(90) 2.353, *p* = .021) levels than the control group (**Table 3**). Moreover, with respect to the cut-off score of the TAS-20 scale, 15 (33.3%) participants in the SPD group, and 9 (19.1%) participants in the control group were identified as having severe alexithymic traits.

**Table 2:** Descriptives of variables.

Variables	TAS-20	DIF	DDF	EOT	FERT Happiness	FERT Sadness	FERT Fear	FERT Anger	FERT Surprise	FERT Disgust	FERT Neutral	BAI	BDI
M	51.68	16.27	13.33	22.07	6.65	4.66	3.54	6.01	5.65	5.31	5.76	11.86	12.29
*SD*	1.14	.67	.36	.38	.10	.19	.18	.12	.16	.15	.19	1.31	1.10

TAS-20, Toronto Alexithymia Scale total score; DIF, Difficulty in identifying feelings; DDF, Difficulty in describing feelings; EOT, Externally oriented thinking; FERT Happiness, Facial Emotion Recognition Test- Happiness subscale;

FERT Sadness, Facial Emotion Recognition Test - Sadness subscale; FERT Fear, Facial Emotion Recognition Test Fear subscale, FERT Anger, Facial Emotion Recognition Test - Anger subscale;

FERT Surprise, Facial Emotion Recognition Test - Surprise subscale; FERT Disgust, Facial Emotion Recognition Test- Disgust subscale; FERT Neutral, Facial Emotion Recognition Test - Neutral subscale;

BAI, Beck Anxiety Inventory total score; BDI, Beck Depression Inventory total score., M, Mean, SD, Standard Deviation.

**Table 3:** Group differences in scale scores.

Scale	SPD	Control	t	df	p	95% CI	Cohen’s d
	*M(SD)*	*M(SD)*					
BAI	18.866 (14.567)	5.170 (4.182)	6.072	50.91	.0001	[9.17, 18.23]	10.615
BDI	17.688 (12.383)	7.127 (4.623)	5.374	55.58	.0001	[6.62, 14.50]	9.268
TAS-20	57.511 (11.108)	52.319 (10.043)	2.421	90	.017	[.97, 9.83]	10.698

*N*= 92, SPD, Skin Picking Disorder Group.

TAS-20, Toronto Alexithymia Scale total score, BAI, Beck Anxiety Inventory total score; BDI, Beck Depression Inventory total score; *M*, Mean,

*SD*, Standard Deviation; CI, Confidence Intervals for the Mean Differences.

### 3.2 Correlational analyses

Pearson correlation analyses were conducted to explore the associations between participants’ levels of alexithymia, depression, and anxiety, and their ability to facial emotion recognition.

The total score of Toronto Alexithymia Scale (TAS-20) exhibited negative correlations with the Facial Emotion Recognition Test (FERT) subscales, namely the sadness (*r* =-.327, *p*=.001), anger (*r* = -.267, *p*= .010), and surprise (*r* = -.422, *p*<.001). However, there were no significant correlations with fear (*r* =-.198, *p*= .059), happiness (*r* =-.078, *p*= .459), disgust (*r* =-.127, *p*= .228) and neutral (*r* =-.150, *p*= .154) state. Distinctly, the level of difficulties in identifying emotions subscale of the TAS-20 was negatively correlated with the fear subscale of the FERT (*r* =-.277, *p*=.007).

BDI (*r* = .495, *p* <.001) and BAI (*r* = .515, *p* <.001) scores of the participants found to be in a positive correlation with their TAS-20 scores. And therefore, added to further analysis as control variables.

All correlations between the TAS-20, FERT, BDI, and BDA scores, as well as the mean and standard deviations of all the scales and subscales are provided in **Table 4**.

**Table 4:** Correlation coefficients of variables.

Variables	TAS-20	DIF	DDF	EOT	FERT Happiness	FERT Sadness	FERT Fear	FERT Anger	FERT Surprise	FERT Disgust	FERT Neutral	BAI	BDI
TAS-20	1												
DIF	.881**	1											
DDF	.835**	.617**	1										
EOT	.655**	.296**	.468**	1									
FERT Happiness	-.078	-.139	-.001	.010	1								
FERT Sadness	-.327**	-.359**	-.216*	-.143	.293**	1							
FERT Fear	-.198	-.277**	-.186	.072	.155	.293**	1						
FERT Anger	-.267*	-.304*	-.161	-.111	.563**	.402**	.267*	1					
FERT Surprise	-.422**	-.367**	-.423**	-.217*	.380**	.428**	.318**	.460**	1				
FERT Disgust	-.127	-.143	-.100	-.033	.418**	.164	.282**	.402**	.197	1			
FERT Neutral	-.150	-.125	-.115	-.121	.502**	.208*	.375**	.514**	.478**	.411**	1		
BAI	.515**	.609**	.282**	.205	-.288**	-.506**	-.360**	-.443**	-.339**	-.160	-.269**	1	
BDI	.495**	.590**	.288**	.173	-.290**	-.371**	-.297**	-.347**	-.365**	-.234*	-.274**	.813**	1

*p <.05; **p <.01 (2-tailed); *N*= 92.

TAS-20, Toronto Alexithymia Scale total score; DIF, Difficulty in identifying feelings; DDF, Difficulty in describing feelings; EOT, Externally oriented thinking; FERT Happiness, Facial Emotion Recognition Test- Happiness subscale;

FERT Sadness, Facial Emotion Recognition Test - Sadness subscale; FERT Fear, Facial Emotion Recognition Test Fear subscale, FERT Anger, Facial Emotion Recognition Test - Anger subscale;

FERT Surprise, Facial Emotion Recognition Test - Surprise subscale; FERT Disgust, Facial Emotion Recognition Test- Disgust subscale; FERT Neutral, Facial Emotion Recognition Test - Neutral subscale;

BAI, Beck Anxiety Inventory total score; BDI, Beck Depression Inventory total score.

### 3.3 Facial emotion recognition in SPD and alexithymia: group comparisons

To examine differences in facial emotion recognition between individuals with Skin Picking Disorder (SPD) and controls, as well as the impact of alexithymia, a series of ANCOVA tests were conducted. Each analysis tested for the main effects of diagnosis (SPD vs. control controls) and alexithymia levels (low vs. high) on emotion recognition performance while controlling for anxiety and depression. Mean and standard deviations of each factor group can be seen in **Table 5**.

**Table 5:** Means and standard deviations by factor groups.

Factors		Dependent Variables						
Diagnosis	Alexithymia	FERT Happiness *M(SD)*	FERT Sadness *M(SD)*	FERT Fear *M(SD)*	FERT Anger *M(SD)*	FERT Surprise *M(SD)*	FERT Disgust *M(SD)*	FERT Neutral *M(SD)*
SPD	Low	6.30 (1.37)	4.47 (1.74)	3.50 (2.06)	5.60 (1.35)	5.73 (1.51)	5.10 (1.42)	5.23 (1.83)
	High	6.33 (1.35)	3.00 (1.96)	1.93 (1.44)	5.27 (1.71)	4.00 (1.89)	4.80 (2.24)	4.13 (2.72)
Control	Low	6.97 (.16)	5.39 (1.46)	4.05 (1.39)	6.55 (.55)	6.26 (1.00)	5.74 (1.11)	6.66 (.58)
	High	7.00 (.00)	5.00 (1.80)	4.22 (1.56)	6.33 (.71)	5.56 (1.24)	5.11 (1.17)	6.44 (1.33)

SPD: Skin Picking Disorder Group; FERT Happiness: Facial Emotion Recognition Test- Happiness subscale; FERT Sadness: Facial Emotion Recognition Test - Sadness subscale; FERT Fear: Facial Emotion Recognition Test - Fear subscale:, FERT Anger: Facial Emotion Recognition Test - Anger subscale, FERT Suprise: Facial Emotion Recognition Test - Suprise subscale, FERT Disgust: Facial Emotion Recognition Test- Disgust subscale, FERT Neutral: Facial Emotion Recognition Test - Neutral subscale; M: Mean, SD: Standard Deviation.

Although the TAS-20 evaluates three separate cognitive aspects of alexithymia, research has shown that the composite TAS-20 score offers a more complete depiction (8). As a result, the total TAS-20 score was used as a grouping variable in the following analyses. Additionally, since participants with SPD often have co-occurring psychiatric conditions and their scores were significantly higher than those of the control group, the analyses controlled for the levels of anxiety and depression among the participants.

The assumption of homogeneity of regression slopes was tested by including interaction terms between the covariates and grouping variables in the ANCOVA models. None of the interaction effects were statistically significant (all p-values >.05), indicating that the relationship between the covariates and the dependent variables did not differ across groups, thus satisfying the assumption.

The analyses did not reveal significant group differences in recognizing happiness, sadness, anger, or disgust. For happiness, Levene’s test was significant, F(3, 88) = 9.33, p <.001, indicating a violation of the homogeneity of variances assumption. Neither SPD diagnosis (F(1,86) = .512, *p*= .476, partial η²= .006) nor alexithymia (F(1,86) = 1.505, *p* = .223, partial η²= .017) had a significant effect, and their interaction was also not significant (F(1,86) = 1.015, *p*= .317, partial η²=.012). Similarly, for sadness, Levene’s test was not significant, F(3, 88) = 0.57, *p* = .638. No significant main effects were found for SPD (F(1,86) = 1.250, *p*= .267, partial η²= .014) or alexithymia (F(1,86) = .737, *p*= .393, partial η²= .008), nor was there a significant interaction (F(1,86) = .010, *p* = .922, partial η²= .000).

For anger recognition, Levene’s test was significant, F(3, 88) = 8.61, p <.001. SPD (F(1,86) = 1.093, *p*= .299, partial η²=.013) and alexithymia (F(1,86) = 0.168, *p*= .683, partial η²=.002) did not show significant effects, nor did their interaction (F(1,86) = 1.037, *p*= .311, partial η²=.012). Similarly, recognition of disgust was unaffected by SPD (F(1,86) = .348, *p*= .557, partial η²= .004), alexithymia (F(1,86) = .525, *p*= .471, partial η²= .006), or their interaction (F(1,86) = 0.480, *p* = .490, partial η²= .006).

While SPD and alexithymia did not significantly influence the recognition of these emotions, anxiety significantly predicted sadness recognition (F(1,86) = 8.283, p = .005, partial η²= .088) and anger recognition (F(1,86) = 5.702, *p* = .019, partial η²= .062). However, depression did not significantly predict recognition of sadness (F(1,86) = 1.095, *p* = .298, partial η²= .013), anger (F(1,86) = 0.065, *p*= .799, partial η²= .001), or disgust (F(1,86) = 2.134, *p* = .148, partial η²= .024).

The SPD group exhibited significant deficits in recognizing fear and neutral expressions compared to controls. Levene’s test indicated that the assumption of homogeneity of variances was met for fear expressions (F(3, 88) = 2.71, p = .050), but violated for neutral expressions (F(3, 88) = 16.87, p <.001). A significant main effect of diagnosis was found for fear recognition (F(1,86) = 5.159, *p*= .026, partial η²= .057), indicating that individuals with SPD had more difficulty recognizing fear than those in the control group. However, alexithymia (F(1,86) = 1.649, *p*= .202, partial η²= .019) and the interaction between SPD and alexithymia (F(1,86) = 2.856, *p*= .095, partial η²= .032) were not significant. For neutral expressions, both SPD (F(1,86) = 21.488, *p* <.001, partial η²= .200) and alexithymia (F(1,86) = 5.317, *p*= .024, partial η²= .058) significantly influenced recognition performance, suggesting that individuals with SPD and those with higher alexithymia levels had greater difficulty in recognizing neutral expressions. However, the interaction between SPD and alexithymia was not significant (F(1,86) = 3.283, *p*= .073, partial η²= .037). Neither anxiety (F(1,86) = 0.651, *p*= .422, partial η²= .008) nor depression (F(1,86) = .340, *p*= .561, partial η²= .004) significantly predicted fear recognition, and neither predicted neutral expression recognition (F(1,86) = 1.705, *p*= .195 partial η²= .019; F(1,86) = .034, *p* = .853, partial η²= .000).

The ability to recognize surprise was significantly impaired in both SPD and alexithymia groups. Levene’s test was significant for surprise expressions, F(3, 88) = 4.36, p = .007. A main effect of SPD was found (F(1,86) = 6.390, *p*= .013), indicating that individuals with SPD had greater difficulty recognizing surprise compared to controls. Additionally, alexithymia significantly influenced surprise recognition (F(1,86) = 10.413, *p*= .002), suggesting that individuals with higher alexithymia levels were also impaired in recognizing surprise. However, the interaction between SPD and alexithymia was not significant (F(1,86) = 2.311, *p*= .132). Neither anxiety (F(1,86) = .676, *p*= .413) nor depression (F(1,86) = .297, *p*= .587) significantly predicted recognition of surprise.

## 4 Discussion

The primary aim of this study was to investigate how emotion recognition abilities vary based on levels of alexithymia and the presence of SPD, both independently and in interaction. To achieve a more comprehensive understanding, we also accounted for the potential confounding effects of anxiety and depression, given their high prevalence in patients with SPD (49) and their known association with emotion recognition performance (50). The findings reveal distinct patterns in emotion recognition among individuals with SPD and those with varying levels of alexithymia, highlighting both shared and unique challenges associated with these conditions.

For the emotion of fear, a significant difference was observed between individuals with SPD and controls, indicating that those with SPD have more difficulty recognizing fear. On the other hand, alexithymia levels alone did not show a significant relationship with the recognition of fear, nor was there a significant interaction between SPD and alexithymia levels. These findings indicate that challenges in recognizing fear might be more specifically associated with the presence of SPD rather than a deficit in emotional processing linked to alexithymia. The difficulties in fear recognition observed in individuals with SPD could stem from broader social cognitive deficits beyond just emotion recognition. For instance, impairments in understanding others’ mental states, such as recognizing and interpreting the intentions, beliefs, and emotions of others, could contribute to these challenges (51). This broader perspective suggests that focusing solely on alexithymia might overlook other critical aspects of social cognition that are affected in SPD.

When evaluating the recognition of surprise, significant differences were found for both SPD diagnosis and levels of alexithymia, indicating that each condition is independently linked to difficulties in recognizing this emotion. However, no significant interaction effect was observed between SPD and alexithymia, suggesting that these factors do not combine to increase difficulties in recognizing surprise. Beyond difficulties in processing their own emotions, individuals with SPD may face additional challenges in recognizing surprise due to specific factors associated with the disorder, such as attentional biases (52), or more generalized deficits in theory of mind and the recognition of nonaffective social cues (53) which are also observed in OCD-related disorders.

Similarly, for neutral expressions, significant differences were observed for both SPD diagnosis and alexithymia levels, implying that both factors are independently associated with difficulties in interpreting these expressions. Again, no significant interaction effect between SPD and alexithymia was detected. Similar to surprise expressions, which can convey a range of emotions like happiness or fear (54), neutral expressions may be especially challenging to interpret due to their ambiguity. This ambiguity can result in biases, leading individuals to perceive emotions that are not present or struggle to detect subtle facial cues (32). Research has shown that higher levels of self-reported childhood trauma, including sexual and emotional abuse and physical neglect, which are linked to increased alexithymia (55), are also associated with greater misinterpretations of neutral facial expressions, often interpreting them as anger, sadness, or contempt (56). Furthermore, research suggests that individuals with body-focused repetitive behavior disorders may exhibit a tendency to disengage from emotional threat cues (57). If these individuals are prone to interpreting neutral stimuli negatively, as indicated by Passardi et al. (56), they may similarly disengage from neutral stimuli. This tendency could result in inadequate processing, leading to inaccuracies in accurately interpreting these expressions. This tendency to disengage from perceived threat cues may reflect underlying avoidant defense mechanisms activated in response to emotional stress (24, 25), and may function as an strategy to minimize the psychological impact of distressing stimuli (58).

In contrast to the findings for fear, surprise, and neutral expressions, no significant differences were observed for the recognition of other emotions, such as happiness, sadness, disgust, and anger, in relation to SPD, alexithymia, or their interaction. This is consistent with studies in the literature, such as Starita et al. (59), which found no differences between high and low alexithymia groups when identifying happy or disgusted facial expressions. On the other hand, Prkachin et al. (60) found that individuals with high levels of alexithymia were less accurate in matching sad, angry, and fearful faces with their corresponding facial expressions, but showed no significant difference compared to individuals with low alexithymia when it came to matching happy, disgusted, or surprised facial expressions. Additionally, those with high alexithymia were capable of correctly identifying all facial expressions in a non-timed setting and rated the intensity of happy and disgusted expressions similarly to the low alexithymia group. It is also noteworthy that among healthy individuals, alexithymia has been linked to difficulties in identifying others’ emotional expressions, particularly when the stimuli are presented for longer durations and with high intensity (61). These contradictory findings suggest that the relationship between alexithymia and emotion recognition is not straightforward and may depend on multiple factors, including the specific emotions being assessed, the methods used to measure emotion recognition (e.g., speeded vs. non-speeded tasks), and the context in which the emotions are presented. Additionally, the absence of SPD-related differences in our sample might be influenced by variables such as age; for example, research indicates that the association between alexithymia and non-suicidal self-injury behavior is more pronounced in younger populations, whereas the link between alexithymia and risky drinking—a different form of maladaptive coping—is stronger in older individuals (62). Future research should explore these variables further to clarify these relationships.

One limitation of the study is that it concentrated mainly on static emotional expressions, which do not frequently occur in everyday situations. In daily life, people typically need to recognize emotions that change dynamically and are expressed at different levels of intensity, making real-world emotional interpretation more complex (59). Additionally, the study’s cross-sectional design prevents any conclusions about causality between variables, limiting our understanding of how these factors may influence each other over time. Another limitation is that our sample consisted of participants who were seeking medical help, which suggests varying levels of awareness regarding their condition. As a result, the findings may not be representative of all individuals with SPD, particularly those who are not needing any medical treatment. Furthermore, reliance on self-report measures introduces potential biases, such as inaccurate self-assessment, which could affect the accuracy of the findings (63). Last but not least, the violation of the homogeneity of variances assumption in several ANCOVA models, as indicated by significant Levene’s test results, should be acknowledged as a limitation. Although the groups in the design were approximately equal in size—an aspect known to reduce the impact of variance heterogeneity on Type I error rates (64), this violation may still compromise the accuracy and robustness of the results.

On the other hand, the study’s methodological rigor was strengthened by the exclusion of participants in the SPD group with a history of psychiatric treatment within the previous year, ensuring that the potential confounding effects of psychotropic medications on emotion recognition were minimized. Moreover, the *a priori* power analysis confirmed that the sample size met the required threshold for detecting medium effect sizes with adequate statistical power, further reinforcing the reliability of the findings. These methodological considerations enhanced the validity of the study and ensured that the observed effects are not driven by sample size limitations or uncontrolled confounding factors.

Overall, this study advances the field by filling gaps related to the complexities of emotion recognition in SPD and alexithymia, offering a more detailed understanding of how these conditions are associated with emotional and social cognitive functions. By highlighting both the shared and distinct challenges of these conditions and accounting for various confounding factors, the study provides a strong foundation for future research to explore these relationships further, especially in more naturalistic and dynamic settings. Understanding that SPD and alexithymia impact emotion recognition through different mechanisms suggests that treatments should be tailored accordingly. As the field continues to grow, building on these findings will be crucial in creating comprehensive, individualized treatment strategies that better address the complexities of emotional and social cognitive functioning in clinical populations. Individuals with SPD who also exhibit elevated alexithymia may benefit from interventions that go beyond symptom management and target foundational deficits in emotion processing and social cognition. For example, mentalization-based therapies (65), which aim to improve individuals’ capacity to understand their own and others’ mental states, may be particularly effective in enhancing emotional awareness. Similarly, computer-based emotion recognition training programs, which have been used in populations with social cognition deficits, could offer accessible and targeted ways to improve facial emotion recognition abilities (e.g. 66, 67). Integrating such approaches into standard therapeutic protocols—such as cognitive-behavioral therapy (CBT) for body-focused repetitive behaviors—may enhance treatment outcomes by addressing underlying emotional and cognitive mechanisms. Further clinical research is needed to examine the effectiveness of these interventions and to assess whether enhancing social-emotional functioning contributes to reductions in symptom severity and improvements in quality of life.

## Data Availability

The raw data supporting the conclusions of this article will be made available by the authors, without undue reservation.
